# Association between parity and adverse maternal and neonatal outcomes: a population-based cross-sectional study

**DOI:** 10.3389/fmed.2025.1697655

**Published:** 2025-11-24

**Authors:** Sisi Li, Jian Li, Liyan Du, Xinling Wang, Yanshang Zhang, Yuange Xiao, Chunying Pan, Yan Huo

**Affiliations:** 1 Department of Obstetrics and Gynecology, Hebei General Hospital, Shijiazhuang, Hebei, China; 2 Department of General Medical, Hebei General Hospital, Shijiazhuang, Hebei, China; 3 Department of Information Management, Hebei Center for Women and Children’s Health, Shijiazhuang, Hebei, China; 4 Department of Internal Medicine of Traditional Chinese Medicine, The Second Hospital of Hebei Medical University, Shijiazhuang, Hebei, China

**Keywords:** parity, maternal outcomes, neonatal outcomes, pregnancy outcomes, cross-sectional study

## Abstract

**Objective:**

This study aimed to comprehensively assess the association between parity and adverse maternal and neonatal outcomes among Chinese women, particularly across different age and educational strata, in the context of recent changes in China’s birth policy.

**Methods:**

This population-based cross-sectional study analyzed data from 451,002 women who delivered at 22 hospitals in 10 cities of Hebei Province, China, between 2013 and 2022. Participants were categorized into three parity groups: the nulliparous (parity = 0) group, primiparous (parity = 1) group, and multiparous (parity ≥ 2) group. Temporal trends in parity composition and maternal age were analyzed using a joinpoint regression analysis to identify significant transition points. Multivariable logistic regression models were used to assess the independent associations between parity and adverse outcomes, adjusting for potential confounders. Subgroup analyses were conducted by maternal age and educational level to examine effect modifications.

**Results:**

From 2013 to 2022, the proportion of multiparous (parity ≥ 2) women and the average maternal age at delivery increased significantly. The joinpoint analysis revealed notable transitions approximately in 2017 in parity composition and in 2019 for maternal age trends among multiparous women, coinciding with major policy changes. After adjusting for confounders, higher parity (compared to nulliparity) was associated with increased risks of anemia, gestational diabetes mellitus (GDM), placenta previa, preterm delivery, macrosomia, stillbirth, and neonatal death (*p*-trend < 0.001). In contrast, higher parity (compared to nulliparity) was associated with lower adjusted odds of hypertension during pregnancy, malpresentation, cesarean delivery, and low birth weight (*p* < 0.001). Subgroup analyses indicated that advanced maternal age (≥35 years) was associated with a heightened risk of perineal laceration among multiparous (parity ≥ 2) women, while lower educational attainment (primary school or below) attenuated the associations between parity and several adverse outcomes, including gestational diabetes, malpresentation, postpartum hemorrhage (PPH), and stillbirth.

**Conclusion:**

The risk of adverse pregnancy outcomes varies significantly by parity, and these relationships are further modified by maternal age and educational level. Tailored prenatal strategies considering parity, age, and education are essential to mitigate risks and improve maternal and neonatal health.

## Background

The one-child policy implemented by the Chinese government in the 1980s effectively controlled the explosive growth of China’s population. However, after more than 30 years of family planning, it inevitably accelerated the process of population aging. Since then, the Chinese government has adjusted the family planning policy in a timely manner, starting with the “two-child single” policy in November 2013, the “universal two-child” policy in January 2016, and the “three-child policy” in May 2021. With the change in birth policy, a contemporary assessment of parity on adverse maternal and neonatal outcomes among Chinese women is needed. However, only a limited number of studies have examined the association between parity and adverse maternal and neonatal outcomes. The results were also inconsistent. Some studies showed (1–3) that the risk of adverse pregnancy outcomes was higher in nulliparas than in primiparas and multiparas (parity ≥ 1). In contrast, Muniro et al. (4) and Dai et al. (5) discovered that primiparous and multiparous (parity ≥ 1) women were at an elevated risk for adverse pregnancy outcomes, such as gestational hypertension, postpartum hemorrhage, and preterm delivery. The majority of previous studies have compared the association of nulliparas with primiparas and multiparas (parity ≥ 1) regarding pregnancy outcomes, but research examining the associations of primiparas (parity = 1) and multiparas (parity ≥ 2) with pregnancy outcomes remains scarce. Based on 10 years of monitoring data in Hebei Province, China, this study aimed to assess the association of different parities (parity = 0, parity = 1, and parity ≥ 2) with adverse maternal and neonatal outcomes. Previous studies have shown that pregnancies at higher and lower ages were more likely to have adverse maternal and neonatal outcomes than those at the appropriate age (6–9). Additionally, there were differences in the association between maternal education and adverse pregnancy outcomes (10–12). Therefore, this study aimed to comprehensively assess the associations between different parity levels (nulliparas, primiparas, and multiparas) and adverse maternal and neonatal outcomes and to evaluate whether these associations were modified by maternal age and educational level. Using a large, multicenter dataset from Hebei Province, China, we sought to provide evidence-based insights for developing tailored prenatal strategies under China’s current three-child policy.

## Methods

### Ethics approval

The data in this study were anonymized and did not invade patients’ privacy; therefore, subjects could not be identified. The ethics committee of Hebei General Hospital waived the requirement for informed consent from patients due to the retrospective nature of the study. The Declaration of Helsinki’s principles were followed in the methods used in this study. The study design was submitted to and approved by the Ethics Committee of Hebei General Hospital (2024-LW-0206).

### Data collection

**Figure 1:**
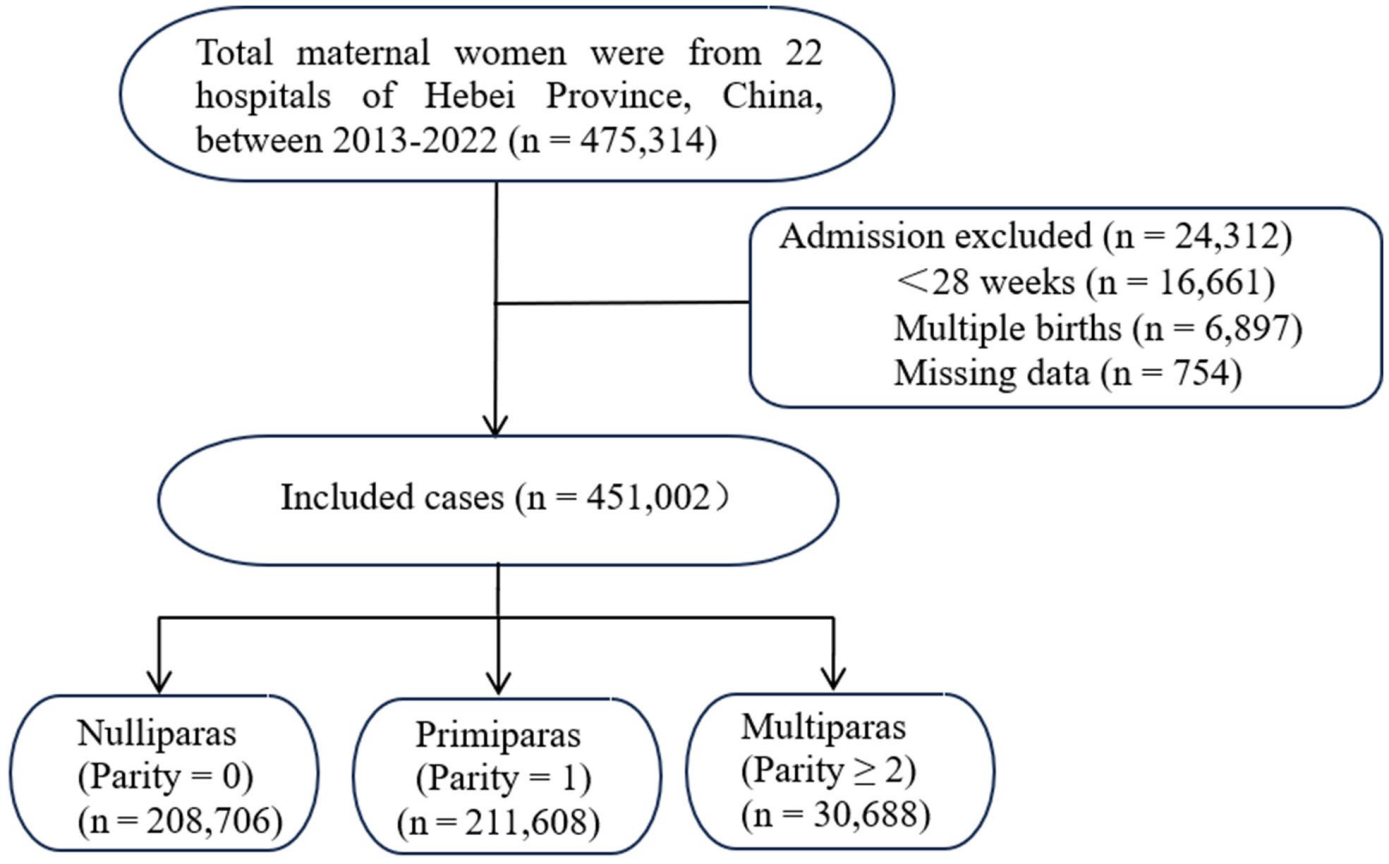
Flowchart of case enrollment.

The study population comprised delivering women from the Hebei Province Maternal Near Miss Surveillance System (HBMNMSS) in the Hebei Center for Women and Children’s Health, which spanned from 1 January 2013 to 31 December 2022. The monitoring system includes 22 monitoring points (hospitals), which were randomly stratified cluster samples and distributed in 10 cities in Hebei Province, China. They covered hospitals of different levels (including tertiary, secondary, and primary hospitals). Data collection was carried out using the “Maternal Case Survey Form,” and all data of discharged maternal women were filled in and reported by uniformly trained obstetricians. The staff of the Hebei Center for Women and Children’s Health checked the data. After excluding pregnancies with gestational age < 28 weeks, multiple gestations, and incomplete data, a total of 451,002 maternal women were included (see Figure 1).

#### Definition

*Exposure variable.* Parity was defined as the number of previous deliveries, excluding the current pregnancy. Women were categorized into three groups: the nulliparous (parity = 0) group, the primiparous (parity = 1) group, and the multiparous (parity ≥ 2) group.

*Outcome variables.* Adverse maternal outcomes in our study included cesarean delivery, anemia, gestational diabetes mellitus (GDM), hypertension during pregnancy (HDP), placenta previa, placental abruption, perineal laceration, postpartum hemorrhage, and obstetric infection. Adverse neonatal outcomes in our study included malpresentation, preterm delivery, macrosomia, low birth weight, low Apgar score, stillbirth, and neonatal death. Cesarean delivery included emergency cesarean delivery and elective cesarean delivery. Based on the World Health Organization (WHO) criteria, anemia was defined as Hb < 110 g/L (13). Gestational diabetes mellitus (GDM) was diagnosed with fasting ≥5.1 mmol/L, 1-h post-prandial ≥10.0 mmol/L, or 2-h post-prandial ≥8.5 mmol/L in a 75-g oral glucose tolerance test (14). Hypertension during pregnancy (HDP) included gestational hypertension, pre-eclampsia, chronic hypertension, chronic hypertension with superimposed pre-eclampsia, and eclampsia (15). Placenta previa was described as a placenta attached to the lower uterine segment lower than the fetal presentation, with the placental margin reaching or covering the cervical os after 28 weeks of gestation (16). Placental abruption was defined as the separation of the placenta from the uterine wall while the fetus remained in the uterine cavity after 20 weeks of gestation (17). Postpartum hemorrhage (PPH) was defined as blood loss of ≥500 mL after vaginal delivery or 1,000 mL after cesarean delivery (18). Obstetric infection is the infection that is diagnosed in the medical record according to the obstetric infection item designed in the “Maternal Case Survey Form,” including uterine incision infections, urinary tract infections, upper respiratory tract infections, thrombotic phlebitis, other systemic infections/sepsis, and puerperal infections. Malpresentation was defined as any fetal presentation other than vertex presentation. A preterm delivery was defined as a delivery that occurred between 28 and 36 + 6 weeks of gestation (19). Macrosomia referred to a birth weight greater than 4,000 g. Low birth weight referred to a birth weight of less than 2,500 g (20). A low Apgar score at 5 min was defined as a total score of ≤7 at 5 min after birth (21). Stillbirth referred to intrauterine fetal demise at more than 27 + 6 weeks of gestation with the fetus weighing 1,000 g or above. Neonatal death referred to those who died at birth or within 7 days of delivery.

*Covariates.* The following covariates were adjusted for in the multivariable models: maternal age, marital status, education level, baby sex, hospital level, number of prenatal care visits, history of cesarean delivery, and history of miscarriage. Based on delivery age, maternal age was categorized into three groups: <20, 20–34, and ≥35 years old. Based on years of education, the education level was categorized into three groups: college and above (≥13 years), middle school (7–12 years), and primary school and below (≤6 years). The number of prenatal care visits referred to the total number of prenatal checkups during pregnancy.

### Statistical analysis

Continuous variables with a normal distribution were presented as mean ± standard deviation (SD), and the one-way ANOVA was used to compare the groups. While continuous variables with skewed distribution were presented as median [interquartile range (IQR)], the Kruskal–Wallis test was used for group comparisons. Statistical data were reported as *n* (%), and the chi-square test (*χ*^2^) was performed to compare groups. With the nulliparous group as a reference, univariable and multivariable logistic regression models were used to examine the effects of different parities on the risk of adverse pregnancy outcomes. After adjusting for potential confounders, subgroup analyses were conducted using multivariable logistic regression models to show the effects of different parities on the risk of adverse pregnancy outcomes in the single age group and the single education level group. With the nulliparous group as a reference, univariable and multivariable logistic regression models were used to examine the effects of different parities on the risk of adverse pregnancy outcomes. The selection of covariates for multivariable adjustment was based on *a priori* knowledge from the literature and the principle of causal temporality. Only variables considered potential confounders that precede the pregnancy outcomes were included. Accordingly, the final models were adjusted for the following covariates: maternal age, marital status, education level, baby sex, hospital level, number of prenatal care visits, history of cesarean delivery, and history of miscarriage. The above statistical analyses were conducted using SPSS software (version 25.0). A joinpoint regression analysis was conducted using the R package (version 4.4.3). Time was treated as discrete annual units in the joinpoint regression analysis, with breakpoints constrained to integer years to enhance.

interpretability and align with the annual nature of the data collection. All statistical tests of hypotheses were two-sided, with a *p*-value of <0.05 being considered statistically significant. GraphPad Prism 6 was used to generate forest plots.

## Results

### Epidemiologic feature

#### Temporal distribution

**Figure 2:**
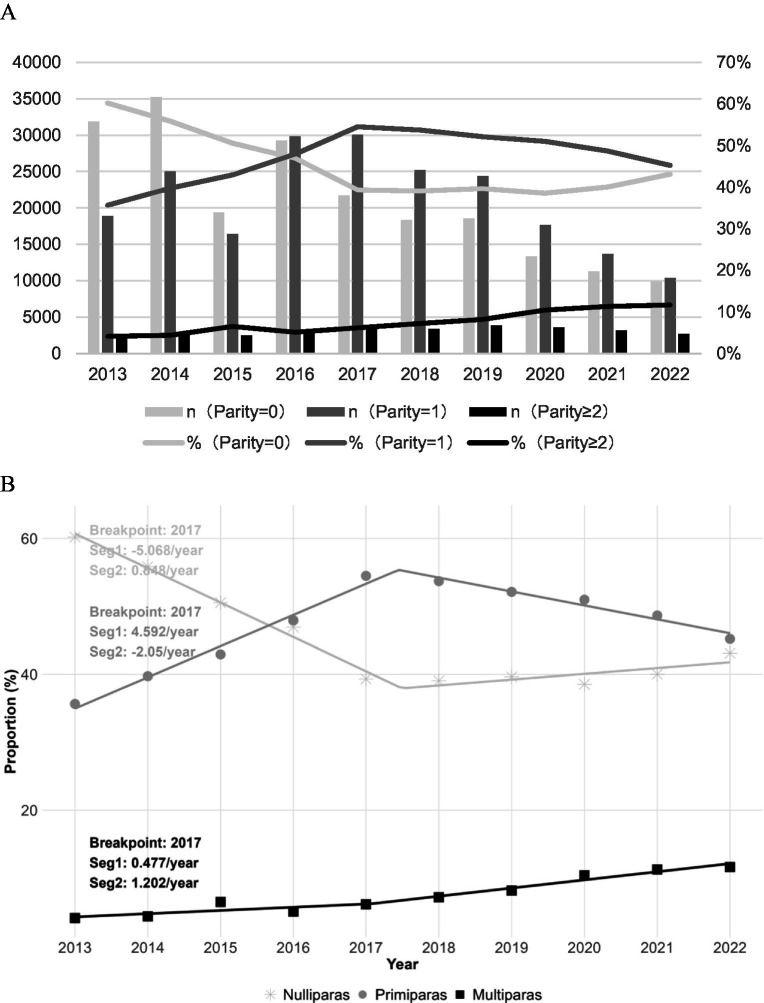
Trends in the number and proportion of women by parity from 2013 to 2022. **(A)** Annual distribution of the number (bars) and proportion (lines) of women in different parity groups: nulliparous (parity = 0), primiparous (parity = 1), and multiparous (parity ≥ 2). **(B)** Temporal trends in the proportion of women across the three parity groups.

Of the 451,002 women studied, 208,706 (46.28%) were nulliparous women (parity = 0), 211,608 (46.92%) were primiparous women (parity = 1), and 30,688 (6.80%) were multiparous women (parity ≥ 2). From 2013 to 2022, the proportion of multiparous women (parity ≥ 2) showed an upward trend (*p* < 0.001) (see Figure 2A). The joinpoint regression analysis using discrete time units revealed significant turning points in the parity composition trends from 2013 to 2022, with all parity groups exhibiting a transition in 2017. For nulliparous women, a distinct transition occurred in 2017, characterized by a significant decreasing trend before the breakpoint (annual change: −5.068, 95% CI: −6.027 to −4.109) followed by a moderate increase thereafter (annual change: 0.848, 95% CI: −0.111 to 1.807). Primiparous women exhibited a similar transition point in 2017, with a substantially increasing trend preceding the breakpoint (annual change: 4.592, 95% CI: 3.819 to 5.365) that reversed to a significant decline afterward (annual change: −2.050, 95% CI: −2.823 to −1.277). Multiparous women also demonstrated a transition in 2017, showing a modest initial increase (annual change: 0.477, 95% CI: −0.085 to 1.039) that accelerated significantly following the breakpoint (annual change: 1.202, 95% CI: 0.640 to 1.764) (see Figure 2B).

#### Age distribution

**Figure 3:**
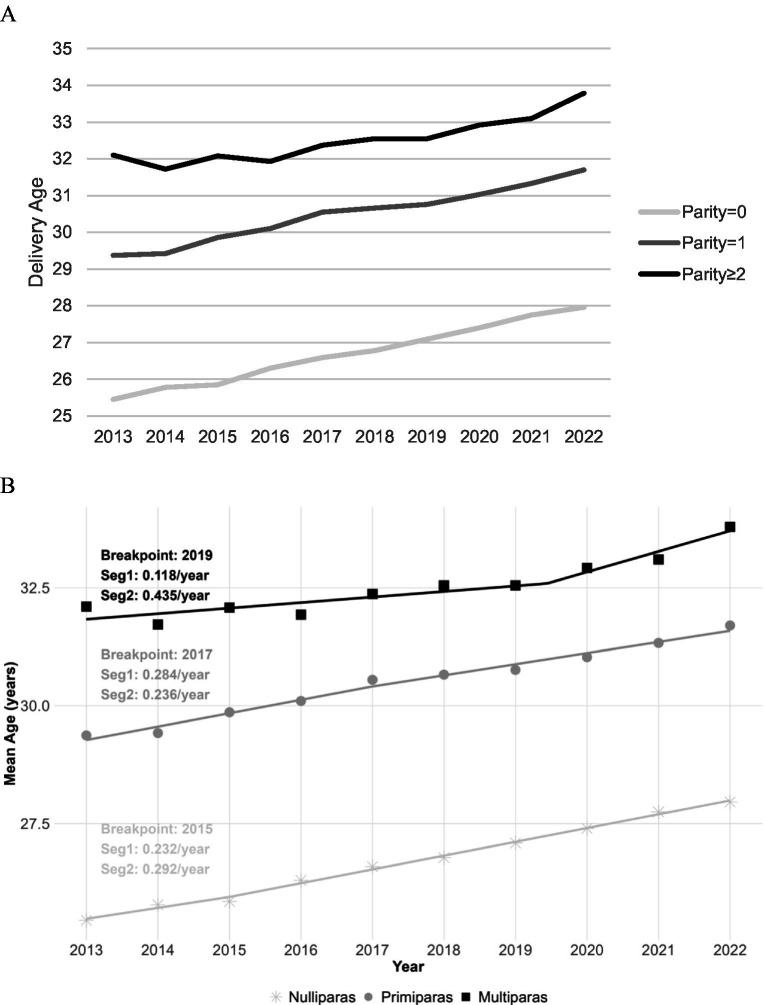
Temporal trends in maternal age at delivery by Papity from 2013 to 2022. **(A)** Annual average delivery age for women stratified by parity. **(B)** Mean age trends for nulliparous, primiparous, and multiparous women.

The average delivery age was 28.70 ± 4.68 years, and as parity increased, the age of delivery exhibited an upward trend. The average delivery ages for the nulliparous (parity = 0), primiparous (parity = 1), and multiparous (parity ≥ 2) groups were 26.42 ± 3.86, 30.39 ± 4.36, and 32.54 ± 4.43, respectively. The average delivery age showed an upward trend from 2013 to 2022 across the three parity groups, respectively. In the nulliparous (parity = 0) group, the average delivery age ranged from 25.45 ± 3.90 years to 27.96 ± 4.05 years (*F*-trend: 904.957, *p* < 0.001). In the primiparous (parity = 1) group, the average delivery age ranged from 29.37 ± 4.44 years to 31.7 ± 4.12 years (*F*-trend: 548.384, *p* < 0.001). In the multiparous (parity ≥ 2) group, the average delivery age ranged from 32.10 ± 5.29 years to 33.79 ± 4.03 years (*F*-trend: 54.913, *p* < 0.001) (see Figure 3A). The joinpoint regression analysis using discrete time units identified distinct temporal patterns in the mean age at delivery across different parity groups from 2013 to 2022. For nulliparous women, a significant transition occurred in 2015, with an annual increase in the mean maternal age accelerating from 0.232 years (95% CI: −0.004 to 0.469) before the breakpoint to 0.292 years (95% CI: 0.266 to 0.318) thereafter. Primiparous women exhibited a breakpoint in 2017, characterized by a deceleration in the rate of increase from 0.284 years annually (95% CI: 0.193 to 0.376) to 0.236 years (95% CI: 0.144 to 0.327). Multiparous women demonstrated the latest transition in 2019, with a marked acceleration in the annual increase from 0.118 years (95% CI: 0.023 to 0.213) to 0.435 years (95% CI: 0.079 to 0.791) following the breakpoint (see Figure 3B).

#### Demographic characteristics of participants

The comparison of maternal and neonatal characteristics by parity is shown in Table 1. Maternal age, marital status, education level, baby sex, hospital levels, gestational age, birth weight, number of prenatal care visits, history of cesarean delivery, and history of miscarriage during the three groups were statistically significant (*p* < 0.001). The proportion of advanced maternal age (≥35 years) increased sharply with parity (nulliparas: 2.65%; primiparas: 17.01%; and multiparas: 31.12%). Higher educational attainment (college and above) decreased with increasing parity (47.92, 31.73, and 17.11%, respectively). A history of cesarean delivery was common among primiparas (44.80%) and multiparas (46.55%). The average times of prenatal care declined with increasing parity. The multiparous group had the highest proportion of male newborns (57.57%).

**Table 1:** Sociodemographic and obstetric characteristics of 451,002 maternal women in Hebei Province, China, from 2013 to 2022.

Items	Nulliparas	Primiparas	Multiparas	*p*
	(Parity = 0)	(Parity = 1)	(Parity ≥ 2)	
	(*n* = 208,706)	(*n* = 211,608)	(*n* = 30,688)	
	*n* (%)	*n* (%)	*n* (%)	
Maternal age (year)				<0.001
≥35	5,522 (2.65)	35,995 (17.01)	9,550 (31.12)	
20–34	198,304 (95.02)	175,127 (82.76)	21,089 (68.72)	
<20	4,880 (2.34)	486 (0.23)	49 (0.16)	
Marital status				<0.001
Single	1,210 (0.58)	721 (0.34)	117 (0.38)	
Married	207,496 (99.42)	210,887 (99.66)	30,571 (99.62)	
Education level				<0.001
College and above	100,012 (47.92)	67,143 (31.73)	5,251 (17.11)	
Middle school	106,169 (50.87)	140,254 (66.28)	23,614 (76.95)	
Primary school and below	2,525 (1.21)	4,211 (1.99)	1,823 (5.94)	
Baby sex				<0.001
Male	105,563 (50.58)	107,751 (50.92)	17,667 (57.57)	
Female	103,143 (49.42)	103,857 (49.08)	13,021 (42.43)	
Hospital levels				<0.001
Primary hospital	1,585 (0.76)	3,496 (1.65)	1,066 (3.47)	
Secondary hospital	147,449 (70.65)	160,493 (75.84)	23,259 (75.79)	
Tertiary hospital	59,605 (28.56)	47,458 (22.43)	6,266 (20.42)	
Home	67 (0.03)	161 (0.08)	97 (0.32)	
Gestational age (weeks)	39.35 (1.64)	39.02 (1.56)	38.78 (1.81)	<0.001
Birth weight (g)	3304.33 (506.56)	3359.61 (509.93)	3349.53 (572.36)	<0.001
Prenatal care visits (number)	7.53 (2.68)	7.07 (2.50)	6.48 (2.545)	<0.001
History of cesarean delivery				<0.001
Yes	0 (0)	94,800 (44.80)	14,285 (46.55)	
No	208,479 (100)	116,808 (55.20)	16,403 (53.45)	
History of miscarriage				<0.001
Yes	44,997 (21.56)	79,586 (37.61)	11,661 (38.00)	
No	163,709 (78.44)	132,022 (62.39)	19,027 (62.00)	

#### Proportion of adverse maternal and neonatal outcomes across parity groups

As shown in Table 2, the differences in the following outcomes among the three parity groups were statistically significant: the proportion of cesarean delivery, anemia, gestational diabetes mellitus, hypertension during pregnancy, placenta previa, placental abruption, perineal laceration, postpartum hemorrhage, obstetric infection, malpresentation, preterm delivery, macrosomia, low birth weight, lower Apgar scores at 5 min, stillbirth, and neonatal death. With an increase in parity groups, the proportion of anemia, placenta previa, preterm delivery, macrosomia, lower Apgar scores at 5 min, and neonatal death showed an upward trend, respectively.

**Table 2:** Adverse maternal and neonatal outcomes across different parities from 2013 to 2022.

Items	Nulliparas	Primiparas	Multiparas	*χ* ^2^	p
	(Parity = 0)	(Parity = 1)	(Parity ≥ 2)		
	*n* (%)	*n* (%)	*n* (%)		
Anemia	59,445 (28.48)	73,360 (34.67)	12,026 (39.19)	2599.911	<0.001
Gestational diabetes mellitus	12,029 (5.76)	14,034 (6.63)	1,667 (5.43)	166.626	<0.001
Hypertension during pregnancy	11,228 (5.38)	10,277 (4.86)	2,096 (6.83)	227.347	<0.001
Placenta previa	509 (0.24)	1,059 (0.50)	366 (1.19)	611.927	<0.001
Placental abruption	567 (0.27)	615 (0.14)	132 (0.43)	23.132	<0.001
Malpresentation	6,474 (3.10)	4,889 (2.31)	850 (2.77)	250.367	<0.001
Preterm delivery	11,801 (5.65)	12,458 (5.89)	2,731 (8.90)	507.371	<0.001
Cesarean delivery	99,386 (47.62)	119,961 (56.69)	17,311 (56.41)	3660.278	<0.001
Perineal laceration	307 (0.51)	239 (0.11)	39 (0.13)	9.478	0.009
Postpartum hemorrhage	2,815 (1.35)	1,851 (0.87)	361 (1.18)	251.374	<0.001
Obstetric infection	1,018 (0.49)	1,060 (0.50)	187 (0.04)	7.929	0.019
Macrosomia	16,208 (7.77)	20,000 (9.45)	3,473 (11.316)	630.491	<0.001
Low birth weight	9,451 (4.53)	8,479 (4.01)	1,753 (5.71)	211.821	<0.001
Lower Apgar at 5 min	441 (0.21)	468 (0.22)	98 (0.32)	14.116	0.001
Stillbirth	1,068 (0.51)	1,023 (0.48)	276 (0.90)	90.088	0.043
Neonatal death	174 (0.08)	187 (0.09)	41 (0.13)	7.738	0.021

#### Association between parity and adverse maternal and neonatal outcomes

**Figure 4:**
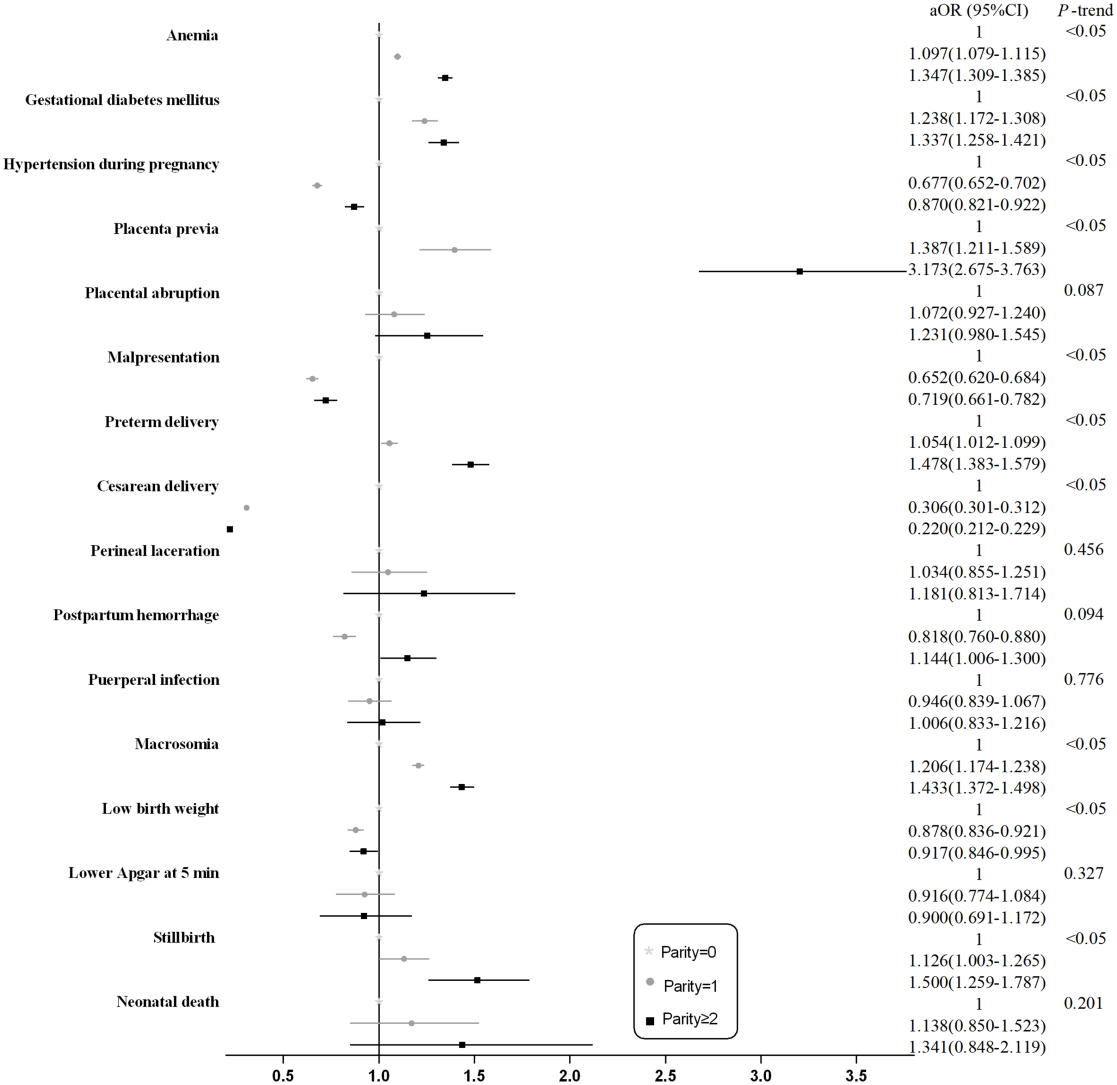
Forest plots of the association between parity and adverse maternal and neonatal outcomes. Compared with nulliparous women. Adjusted by maternal age, marital status, education level, baby sex, hospital levels, number of prenatal care visits, history of cesarean delivery, and history of miscarriage.

After adjusting for potential confounders, women with increasing parity groups showed an increased risk of anemia, gestational diabetes mellitus, placenta previa, preterm delivery, macrosomia, stillbirth, and neonatal death (*p*-trend <0.001). In contrast, primiparous and multiparous (parity ≥ 1) women were at a reduced risk of hypertension during pregnancy, malpresentation, cesarean delivery, and low birth weight (*p* < 0.001). However, there were no statistically significant differences in the effects of placental abruption, perineal laceration, and puerperal infection (see Figure 4).

**Figure 5:**
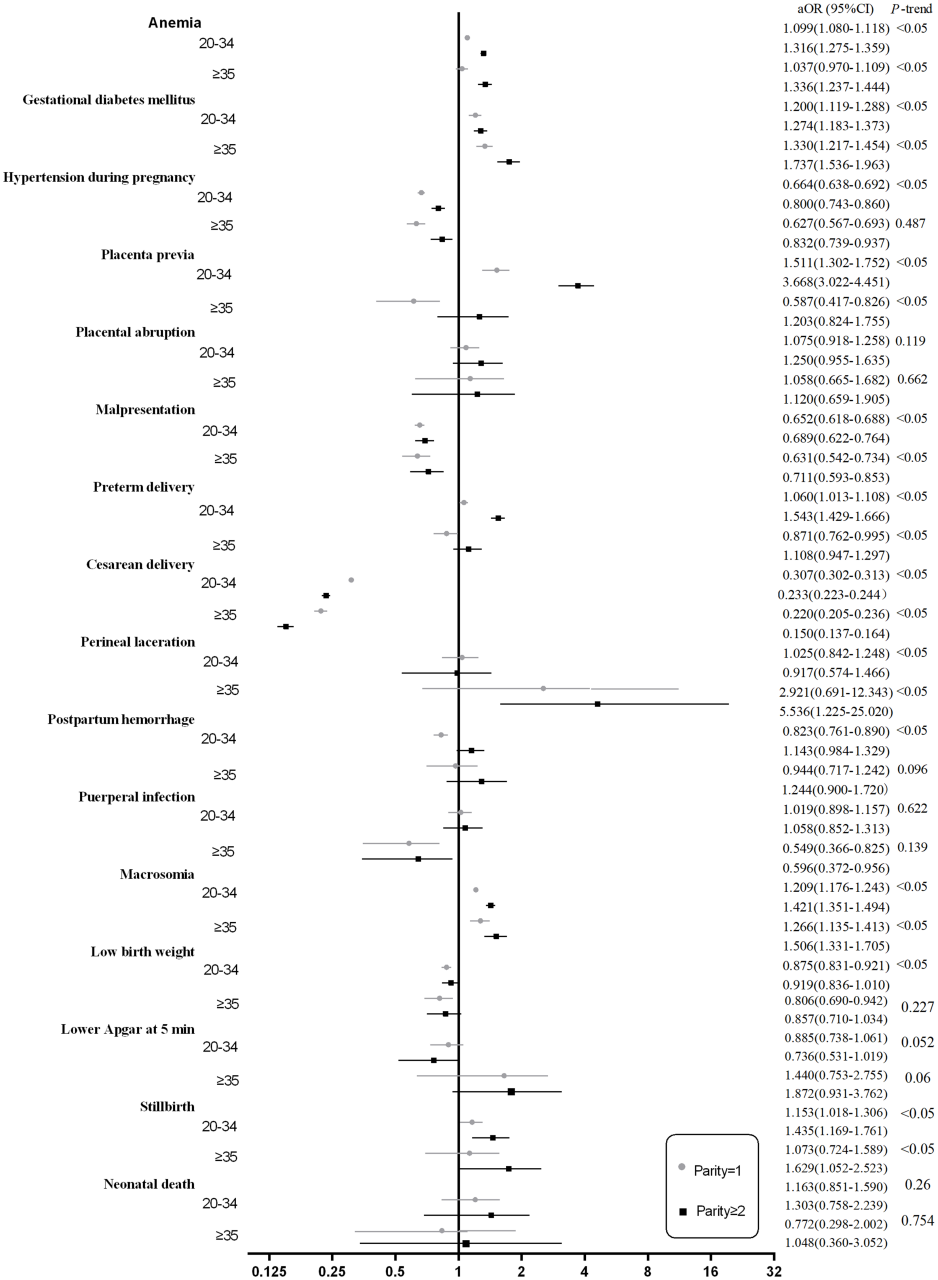
Associations between parity and pregnancy outcomes stratified by age.

**Figure 6:**
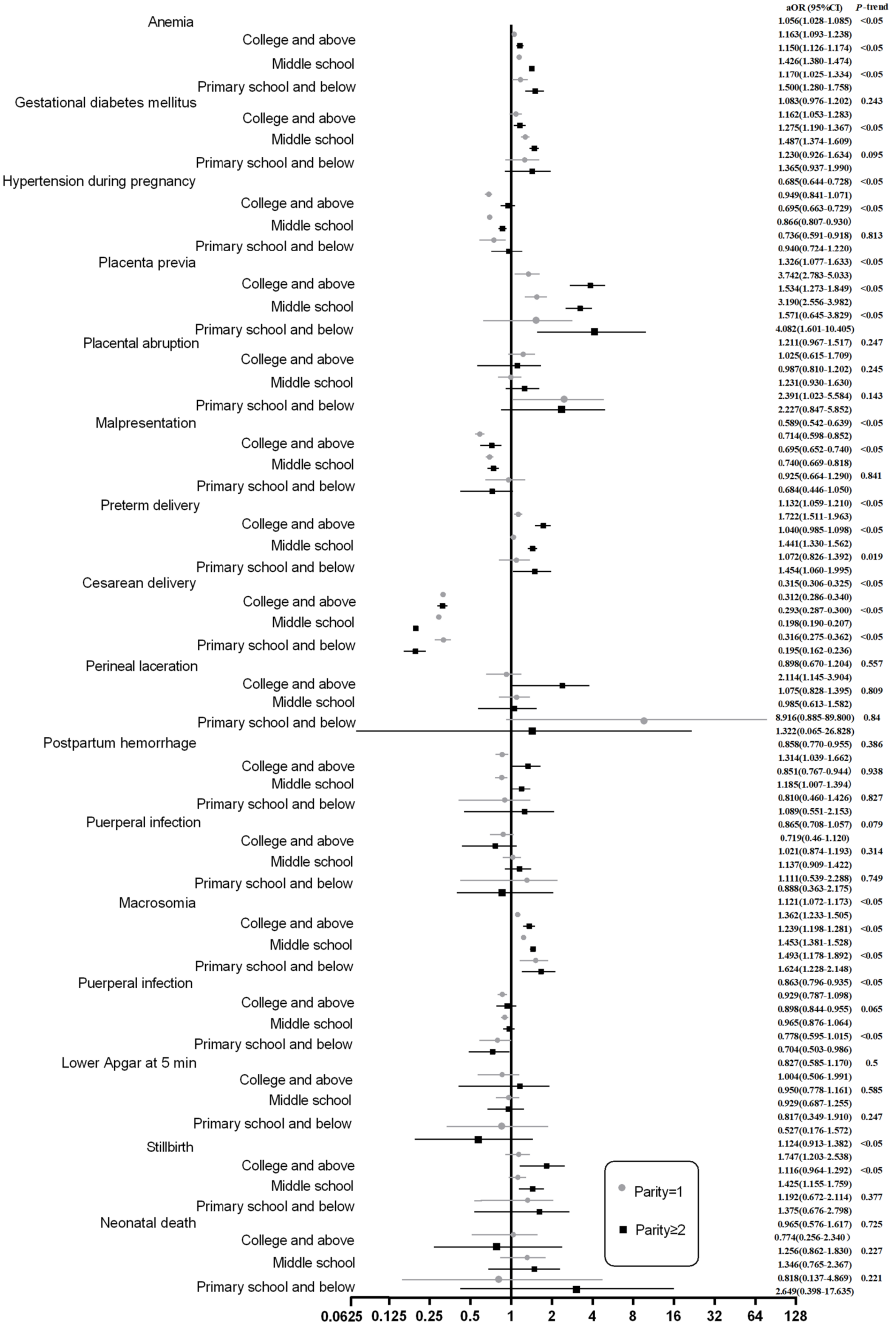
Associations between parity and pregnancy outcomes stratified by education level.

Subgroup analyses were conducted to evaluate the associations between parity and pregnancy outcomes stratified by maternal age and education level (Figures 5,6).

Compared with nulliparous (parity = 0) women. Compared with nulliparous women. Adjusted by marital status, education level, baby sex, hospital levels, number of prenatal care visits, history of cesarean delivery, and history of miscarriage.

*Stratification by age* (Figure 5): In the <20-year-old group, there were very few multiparous women (parity ≥ 2). So when analyzing the associations between parity and pregnancy outcomes stratified by age, we excluded the <20-year-old group. When stratified by maternal age, the overall trends observed in the primary analysis remained largely consistent across the 20–34 and ≥35 years of age groups. However, several notable interactions were identified. Among women aged ≥35 years, multiparity (parity ≥ 2) was associated with a significantly increased risk of perineal laceration (aOR: 5.536, 95% CI: 1.225–25.020). Conversely, in this same age group, primiparous and multiparous women (parity ≥ 1) demonstrated a reduced risk of puerperal infection compared to nulliparous women (aOR: 0.596, 95% CI: 0.372–0.956). Furthermore, parity showed no significant association with the risk of preterm delivery in women aged ≥35 years.

Compared with nulliparous (parity = 0) women. Compared with nulliparous women. Adjusted by maternal age, marital status, education level, baby sex, hospital levels, number of prenatal care visits, history of cesarean delivery, and history of miscarriage.

*Stratification by education level* (Figure 6): Subgroup analysis by educational attainment revealed that the associations between parity and the majority of adverse outcomes were consistent across different education levels. However, among women with an education level of primary school or below, parity was not significantly associated with risks of gestational diabetes mellitus, malpresentation, postpartum hemorrhage, or stillbirth, indicating a potential modifying effect of lower educational attainment on these relationships.

## Discussion

Our findings demonstrated significant shifts in China’s fertility patterns following the implementation of the “two-child” and subsequent “three-child” policies. The synchronized transition points across parity groups approximately in 2017, characterized by reversed trends in nulliparous and primiparous proportions alongside accelerated multiparous growth, suggested that these policy changes substantially influenced reproductive decisions (6). Our observation of a progressive increase in maternal age is in line with the trends reported in previous studies (22, 23). The accelerated increase in age observed among multiparous women after 2019 indicated a lengthening reproductive span and potential compression of fertility timelines (5). The observed trends presented a complex demographic scenario. While policy relaxation enabled higher-order births, it also contributed to delayed childbearing, with multiparous women averaging 32.54 years—substantially older than nulliparous women (26.42 years). This age disparity carried significant clinical implications, as advanced maternal age is strongly associated with adverse pregnancy outcomes (22). The consistent upward trajectory in multiparous maternal age from 32.1 to 33.79 years during 2013–2022 underscored an urgent need for targeted healthcare strategies to address the growing challenges of older multiparity. These patterns reflected an ongoing fertility transition where policy interventions interacted with socioeconomic factors to reshape reproductive behaviors. The findings highlighted the importance of developing comprehensive maternal healthcare services that address the unique needs of an increasingly older pregnant population while maximizing the benefits of fertility policy adjustments.

In this 10-year cross-sectional study of Chinese women, we found that the proportion of multiparous (parity ≥ 2) women exhibited an upward trend from 2013 to 2022, which is in line with the adjustments to birth policy. After adjusting for potential confounders, we found that women with increasing parity groups showed an increased risk of anemia, gestational diabetes mellitus, placenta previa, preterm delivery, macrosomia, stillbirth, and neonatal death (*p*-trend < 0.001). In contrast, primiparous and multiparous (parity ≥ 1) women were at a reduced risk of hypertension during pregnancy, malpresentation, cesarean delivery, and low birth weight (*p* < 0.001).

As one of the adverse pregnancy outcomes, the prevalence of cesarean delivery has increased substantially worldwide. In China, the cesarean delivery proportion increased from 28.8 to 34.9% between 2008 and 2014 (24), which was significantly higher than the World Health Organization (WHO)-recommended rate of 10–15% (25). This study found that the cesarean delivery proportion among primiparous and multiparous (parity ≥ 1) women was higher than that of nulliparous women. However, after adjusting for potential confounders, parity was found to be negatively correlated with cesarean delivery, indicating that the risk of cesarean delivery decreased with higher parity. Stratified analyses by age and education yielded the same findings. Nulliparous women typically experience longer labor duration and were worried about the need for the cesarean section after a failed trial delivery or because they were afraid of the pain of a long labor course, so many nulliparous women requested elective cesarean deliveries without valid medical indications (26). After experiencing vaginal delivery, the labor duration for primiparous and multiparous (parity ≥ 1) women was shorter, and the pain associated with vaginal delivery was more tolerable. Consequently, the inclination to request cesarean delivery was reduced among primiparous and multiparous (parity ≥ 1) women. Dai et al. (5) found that multipara was a risk factor for cesarean delivery, while Alshammari et al. (27) reported no association between parity and cesarean delivery. This discrepancy may be attributed to the differing confounders that were adjusted in each study. Women with a previous history of cesarean delivery were more likely to undergo cesarean delivery in subsequent births (28). However, studies by Dai et al. (5) and Alshammari et al. (27) did not adjust for previous cesarean delivery history, resulting in differences in the results from this study.

Both this study and previous reports (29) suggested that primiparous and multiparous (parity ≥ 1) women were more prone to anemia during pregnancy than nulliparous women. After adjusting for potential confounders, higher parity was associated with increased odds of anemia during pregnancy, with the prevalence increasing across parity groups. The elevated risk of anemia during pregnancy in primiparous and multiparous (parity ≥ 1) women may be attributable to inadequate replenishment of iron reserve depletion during previous pregnancies and lactation (29). Additionally, short birth intervals contributed to an increased risk of anemia during pregnancy (30). Birth interval was not investigated in this study; relevant research could be conducted in the future.

In our study, we observed a positive association between parity and the risk of gestational diabetes mellitus, with the frequency of gestational diabetes increasing alongside higher parity. Stratified analyses by age and education yielded similar findings. Studies (31, 32) suggested that high parity was an independent risk factor for gestational diabetes. A study (33) indicated that there was a linear relationship between high parity in postmenopausal women and type 2 diabetes. Furthermore, studies showed that in perimenopausal women, after adjusting for age, body mass index (BMI), and other factors, the number of births was associated with an increased risk of type 2 diabetes (33) and dyslipidemia (34, 35). This suggested that high parity may elevate women’s long-term risk of chronic diseases.

Hypertension during pregnancy was a leading cause of maternal morbidity and mortality worldwide (36). Our analysis demonstrated an independent association between parity and hypertension during pregnancy. Primiparous and multiparous (parity ≥ 1) women had lower adjusted odds of hypertension during pregnancy, which is consistent with previous studies (37–39). Although the physiological mechanism of primiparity and multiparity as a protective factor for hypertension during pregnancy was not well-defined, Suzuki et al. (40) and Prefumo et al. (41), using Doppler ultrasound, found that parity had a significant impact on the resistance index of the uterine artery and the blood flow wave form of the uterine artery, suggesting that the previous pregnancy may have made the mother’s uterus and blood vessels retain some permanent structural changes (42), and these physiological structural changes also reduced the impedance of hemodynamics in women during subsequent pregnancies, which was more conducive to the exchange of substances between the mother and the fetus.

This study indicated that primiparas and multiparas (parity ≥ 1) were associated with an increased risk of delivering macrosomia and a decreased risk of delivering low birth weight, consistent with the findings of prior studies (3, 43, 44). This might be related to reduced uterine placental blood flow and a smaller uterine cavity in nulliparous women (45, 46).

As a global issue, preterm delivery was considered one of the primary health indicators of a country (47). Over the past two decades, the prevalence of preterm delivery has been increasing in the majority of countries and has affected approximately 11% of all births worldwide (48). We found higher parity was associated with an increased odds of preterm delivery. Vaswani et al. (49) found that the risk of preterm delivery was linearly correlated with parity, increasing with higher parity levels. Their findings were consistent with our study. A possible mechanism was that previous pregnancies led to endometrial injury, resulting in dysplasia of the decidua during subsequent pregnancies. This condition might increase the levels of cytokines and enzymes that degrade the extracellular matrix, ultimately inducing uterine contractions and leading to preterm delivery (50). However, in our study, subgroup analyses by age revealed no association between parity and preterm delivery in women over 35 years. The absence of a significant association between parity and preterm delivery in women aged ≥35 years diverges from our overall findings and those of some previous studies (49). This suggests that in advanced maternal age, the well-established age-related risk factors for preterm birth may overshadow the additional risk contributed by parity itself.

In our study, in a univariable analysis, multiparity (parity ≥ 2) was associated with placental abruption, with an odds ratio (OR) of 1.289 (95% CI: 1.229, 1.353). However, after adjusting for potential confounders, no significant association was found between parity and placental abruption. Stratified analyses by age and education yielded similar results. The etiological mechanisms underlying placental abruption remain incompletely elucidated. Sheiner et al. found that grandmultiparity (defined as more than five deliveries) was independently correlated with the occurrence of preterm placental abruption (51). The possible reason was that the study target and the parity group were different. The target of Sheiner et al.’s study was preterm placental abruption. Perineal laceration was the most common complication associated with vaginal delivery (52). In this study, the proportion of perineal laceration in primiparas was higher than in multiparas. However, after adjusting for potential confounders, no significant association was found between parity and perineal laceration. Stratified analyses by age and education revealed that, in the group aged ≥ 35 years or with a college education and above, a multiparous (parity ≥ 2) woman was a risk factor for perineal laceration. Multiparas, particularly those with a parity ≥ 2, often experienced rapid labor during childbirth. Consequently, the perineum may not have undergone sufficient dilation, and the flexibility of the pelvic floor and perineal tissue tends to decrease with increasing parity and age. This may explain why, in the older women’s group, a parity ≥ 2 was associated with a higher risk of perineal laceration.

We found that there was no association between parity and puerperal infection. However, stratified analyses by age indicated that primiparous and multiparous (parity ≥ 1) women who were ≥35 years old had a higher risk of puerperal infection. A few previous studies have examined the relationship between parity and puerperal infection. A study conducted by Yuan et al. (53) that involved 268,311 women showed that parity was not associated with puerperal infection, which is consistent with our findings. Nevertheless, additional studies on the association between parity and puerperal infection were needed. Our analysis demonstrated that the proportion of placenta previa increased with higher parity, which is consistent with Gilliam et al.’s, study (54). Endometrial injury was the primary cause of placenta previa. Abortion, delivery, and cesarean delivery might lead to endometrial injury, and the severity of this injury tended to increase with higher parity. The proportion of postpartum hemorrhage was higher in the multiparous (parity ≥ 2) group. Vaswani et al. (49) found that the risk of postpartum hemorrhage was linearly correlated with parity, increasing with each additional pregnancy. This might be attributed to the excessive elongation and contraction of the myometrium caused by multiple pregnancies and childbirth. Factors such as inadequate uterine rehabilitation, poor repair of the myometrium, and diminished contractile ability could lead to weak postpartum uterine contractions, making individuals more susceptible to post-delivery bleeding. At the same time, as delivery times increase, the number of uterine operations might also rise, leading to a higher prevalence of placental adhesion, implantation issues, or placenta previa. Consequently, there was an increased likelihood of experiencing uterine weakness or postpartum hemorrhage due to placental factors. Witkop et al. (55) observed that the proportion of malpresentation in nulliparous women was twice as high as in primiparous and multiparous (parity ≥ 1) women. This finding aligns with our results, which may be attributed to the more relaxed muscle tone in the uterus and abdominal wall of primiparous and multiparous (parity ≥ 1) women (56). Our data indicated that stillbirth was independently associated with parity. The risk of stillbirth was 1.126 times higher in primiparas (parity = 1) (95% CI 1.003–1.265) and 1.500 times higher in multiparas (parity ≥ 2) (95% CI 1.259–1.787) than in primiparas. A study involving a total sample of 28,626 births also demonstrated that high parity accelerated the rate of stillbirths (57).

Furthermore, the modifying effect of education level highlights the complex interplay between biological and socioeconomic factors. The lack of significant associations between parity and several outcomes (GDM, malpresentation, PPH, and stillbirth) among women with the lowest education level (primary school or below) suggests that the profound socioeconomic disadvantages and potentially limited access to prenatal care in this group may be such powerful determinants of adverse outcomes that they mask the independent effect of parity (10–12). This underscores the necessity of addressing social determinants of health in public health strategies aimed at improving maternal and neonatal outcomes, particularly in vulnerable populations.

We investigated the interaction effects of education and parity on pregnancy outcomes, filling a gap in this area. This study was a decade-long, multi-center, cross-sectional retrospective survey involving 22 hospitals in Hebei Province, China. A total of 451,002 delivery records were included in the analysis. The sample size was substantial and representative. During the data collection process, the questionnaire data were meticulously checked and cleaned repeatedly. The authenticity and reliability of the results reflect the associations of parity with adverse pregnancy outcomes. However, several limitations should be considered when interpreting our findings. First, data on important potential confounders were unavailable, including pre-pregnancy body mass index, interpregnancy intervals, detailed socioeconomic status, occupational exposures, and behavioral factors such as smoking and nutrition. The absence of these variables may have resulted in residual confounding. Second, as a hospital-based study, our findings may not be fully generalizable to community settings or other regions with different healthcare systems. Third, the observational cross-sectional design precludes causal inference. Future prospective studies with more comprehensive data collection are warranted to validate our findings and explore the underlying mechanisms.

## Conclusion

In this large multicenter study, we observed that parity has distinct associations with various adverse pregnancy outcomes. Specifically, higher parity (compared to nulliparity) was associated with increased risks of anemia, gestational diabetes mellitus, placenta previa, preterm delivery, macrosomia, stillbirth, and neonatal death, while it was associated with decreased risks of hypertension during pregnancy, malpresentation, cesarean delivery, and low birth weight. These relationships were further modified by advanced maternal age and lower educational attainment. Our findings underscore the importance of developing tailored prenatal strategies that consider a woman’s parity, age, and education level to mitigate specific risks and improve maternal and neonatal health. For instance, multiparous women may benefit from enhanced screening for anemia, gestational diabetes, and placenta previa, along with closer monitoring for preterm delivery. Nulliparous women might require support for vaginal birth preparation, hypertension monitoring, and management of prolonged labor. Women with lower educational attainment could be prioritized for health literacy interventions, prenatal education, and socioeconomic support to address barriers to care. Such risk-stratified approaches may help optimize perinatal care and improve perinatal outcomes in the context of China’s evolving birth policy.

## Data Availability

The original contributions presented in the study are included in the article/supplementary material, further inquiries can be directed to the corresponding authors.
